# A Kinase Duet Performance in the Asymmetric Division of *Drosophila* Neuroblasts

**DOI:** 10.3390/jdb5030007

**Published:** 2017-09-14

**Authors:** Christopher A. Johnston

**Affiliations:** Department of Biology, University of New Mexico, Albuquerque, NM 87131, USA; johnstca@unm.edu; Tel.: +1-505-277-1567

**Keywords:** asymmetric cell division, cell polarity, neural stem cell, neuroblast, partner of inscuteable, myosin, protein kinase

The ability of progenitor stem cells to divide asymmetrically allows for the production of diverse daughter cell fates. This process typically follows the sequential steps of cortical cell polarity and mitotic spindle positioning to ensure unequal distribution of molecular fate specifying factors. For divisions that produce daughter cells that are also of different sizes, an additional requisite is the asymmetric displacement of non-muscle Myosin II and site of cleavage furrow ingression. Previous work had demonstrated that the apical polarity complex (specifically the Partner of Inscuteable, Pins, protein) functions in a spindle-independent pathway for such asymmetric cleavage furrow establishment in *Drosophila* neural stem cells (neuroblasts). New work from the Cabernard lab has now uncovered a molecular pathway for this essential component of cell division asymmetry.

Again employing *Drosophila* neuroblasts as a model system, Tsankova, et al. [1] have revealed a polarity-dependent phoshoregulation of Myosin involving Rho kinase (Rok) and Protein kinase N (Pkn) that control both the spatial and temporal activity of its motor function. The authors provide evidence that phospho-Myosin becomes apically enriched in the early phases of mitosis through the activity of Rok, which itself is enriched by the Pins polarity complex. Later in the cell cycle, Pins generates apically enriched Pkn, which in turn inhibits Rok/phospho-Myosin specifically at the apical surface, thereby leading to the basal Myosin shift characteristic of asymmetric neuroblast divisions. Indeed, the activities of these kinases are integral not only to proper Myosin localization dynamics, but also proper cleavage furrow positioning and daughter size asymmetry. These results were generated from elegant genetic studies using live imaging of neuroblasts expressing a fluorescent Myosin sensor. The model proposed by Tsankova significantly extends our understanding of the molecular determinants underlying a critical element of asymmetric cell division and establish yet another function for the intriguingly multitalented Pins protein.
